# Mobility surveillance as a public health blind spot in long-term care

**DOI:** 10.3389/fpubh.2026.1792509

**Published:** 2026-05-26

**Authors:** Neha Sabharwal

**Affiliations:** Vintage Faire Nursing and Rehab, Modesto, CA, United States

**Keywords:** functional outcomes, long-term care, mobility decline, nursing homes, public health surveillance, skilled nursing facilities

## Abstract

Public health surveillance in long-term care has appropriately prioritized acute adverse events that are discrete, reportable, and survey-sensitive, including falls, infections, pressure injuries, and hospital transfers (1, 2). These indicators capture immediate harm and regulatory risk, yet they only partially reflect resident trajectories. Functional mobility decline is common in institutional settings and is consistently associated with reduced community discharge, higher rehospitalization risk, and longer-term dependency (3–7). Although long-term care systems routinely collect functional data through standardized instruments such as the Minimum Data Set (MDS) 3.0 and the Continuity Assessment Record and Evaluation (CARE) Item Set (8–10), these data are rarely aggregated and interpreted as population-level surveillance signals. The problem is not assessment. It is the absence of surveillance-oriented use of existing function data. This perspective argues that mobility decline should be treated as an underrecognized public health signal in long-term care. It proposes a conservative path forward focused on feasibility, risk adjustment, ethics, and non-punitive use, so mobility trajectories can complement, not replace, current surveillance frameworks.

## Introduction

Long-term care includes nursing homes, skilled nursing facilities, and post-acute rehabilitation environments that together serve a large and growing population of older adults with multimorbidity and functional vulnerability. In the United States, the Centers for Medicare & Medicaid Services (CMS) (1, 2) maintains national quality reporting and public transparency mechanisms for nursing homes through Nursing Home Compare and related quality measure programs. These systems were built to detect harm that is discrete, time-limited, and immediately recognizable, because that is the class of events most visible to residents, families, surveyors, and payers.

Yet many outcomes that matter most to residents and families unfold gradually. Mobility can erode through short walks avoided, transfers performed for speed rather than practice, and a culture that equates safety with stillness. This decline may occur without a sentinel event and can become normalized as expected aging. The literature, however, has repeatedly shown that functional loss is not merely a benign accompaniment to illness. It predicts discharge destination, post-acute trajectory, and downstream health system utilization (3–7).

A critical distinction is therefore required. Long-term care already measures function. What is missing is surveillance. Surveillance is not a single assessment. It is the systematic aggregation, interpretation, and feedback of data to detect patterns and guide response at the population level (11, 12). CDC guidance identifies five core functions of effective surveillance: systematic data collection, ongoing analysis, interpretation, timely dissemination, and a linked organizational response (11). This perspective examines why mobility decline remains largely absent from long-term care surveillance and proposes practical, ethically grounded approaches for integrating mobility trajectories into existing monitoring structures.

### What public health surveillance currently prioritizes

Surveillance frameworks in long-term care focus on outcomes that are immediately consequential, reliably defined, and linked to oversight. Falls, healthcare-associated infections, pressure injuries, and rehospitalizations are tracked because they represent identifiable episodes of harm and are tied to star ratings, public reporting, and reimbursement consequences (1, 2). These metrics are reviewed in quality meetings, reported to leadership, and often incorporated into facility-level performance improvement agendas.

This design is understandable, but it produces a predictable analytic blind spot. Gradual functional decline does not occur as a single reportable event. It is experienced as a sequence of small losses. When surveillance focuses only on discrete harms, organizations may inadvertently treat mobility loss as a clinical detail rather than a system signal. Over time, what is not trended becomes less visible, and what is less visible is less protected.

### Functional assessment exists. Surveillance does not

The absence of mobility surveillance does not imply an absence of mobility measurement. Nursing homes routinely assess function using the MDS 3.0, which includes items capturing self-performance and support needs across mobility and activities of daily living domains (8, 9). Post-acute settings have also relied on the CARE Item Set and related standardized patient assessment instruments intended to harmonize functional measurement across care settings (10). Therapy teams additionally document mobility intensity and performance using validated or semi-validated facility tools, often at higher frequency than required by standardized assessments.

However, the dominant use case for these data is administrative and resident-level, not surveillance-level. MDS data support care planning, compliance, and payment classification. CARE items support cross-setting measurement. Neither is typically deployed as a routine signal of emerging population risk. Evidence suggests that linking standardized assessment data to broader analytics can be feasible and valuable, but requires intentional infrastructure and interpretation (13). The gap, therefore, lies in the translation of assessment data into population monitoring and organizational learning.

### How this proposal differs from existing quality indicators

Existing quality measurement systems include mobility-related items. The CMS Nursing Home Compare long-stay quality measure tracking the percentage of residents whose ability to move independently worsened (MDS Quality Measure 5.3) captures individual-level decline and is publicly reported at the facility level (14). The aged care quality indicator repository and analogous frameworks internationally similarly include function-based performance markers, and mobility-related searches yield numerous potentially relevant results. These are valuable contributions. However, they share a common design logic, producing facility-level summary statistics intended for accountability, benchmarking, and public reporting. They are quality indicators, not surveillance signals.

The distinction matters. A quality indicator reports the proportion of residents who declined over a defined period. A surveillance signal tracks whether that proportion is changing over time, whether it differs across units or shifts, whether changes in staffing or care practice precede changes in trajectory, and whether patterns of decline are inequitably distributed. Surveillance applies a population monitoring lens to detect emerging signals, prompt investigation, and support organizational learning. It does not merely report a number for external comparison. This manuscript does not propose a new quality indicator. It proposes a different analytical use of functional data that facilities already collect, applying the logic of epidemiological surveillance to patterns that quality metrics capture only in cross-sectional snapshot form (11, 12).

### Why this gap matters

Functional decline has meaningful downstream consequences. Studies have documented that older adults frequently experience functional loss in association with acute illness and institutional care, and that such decline predicts readmissions and longer-term dependency (4–7, 15, 16). Functional impairment has also been associated with increased risk of 30-day readmission among Medicare beneficiaries (17). In long-term care settings, mobility loss shapes the probability of community discharge and influences the intensity of caregiver needs after discharge (3, 5).

From a system perspective, mobility decline is not simply a clinical outcome. It is a proxy for the extent to which institutional environments preserve or erode function. When mobility trajectories are not monitored, facilities lack a structured way to detect patterns, compare units, identify inequities, or evaluate whether safety practices inadvertently trade independence for immobility. A surveillance lens would allow organizations to treat mobility decline as an early signal and to target review before decline becomes irreversible.

### Conceptual framework for mobility decline in institutional care

Mobility decline in institutional settings can be understood using established models of disability and function. The disablement process framework described by Verbrugge and Jette (18) emphasizes the interaction of pathology, impairment, functional limitation, and disability, shaped by intra-individual and extra-individual factors. The World Health Organization’s International Classification of Functioning, Disability and Health (ICF) (19) similarly locates functioning at the intersection of health conditions, activity limitations, participation restrictions, and contextual influences.

Within long-term care, individual-level vulnerability interacts with facility-level context. Environmental constraints, staffing pressures, and care routines can limit opportunities for safe movement. Risk-averse responses to falls may restrict ambulation and inadvertently accelerate deconditioning. These dynamics are amplified when therapy episodes end and the responsibility for maintenance is diffused across teams with competing priorities.

Surveillance functions as a systems intervention point. By aggregating functional trajectories and flagging emerging decline, population-level monitoring can trigger interdisciplinary review. It does not prescribe a single intervention. It creates visibility, enabling teams to ask why decline is occurring, whether it is goal-concordant, and what modifiable factors may be contributing.

**Figure 1:**
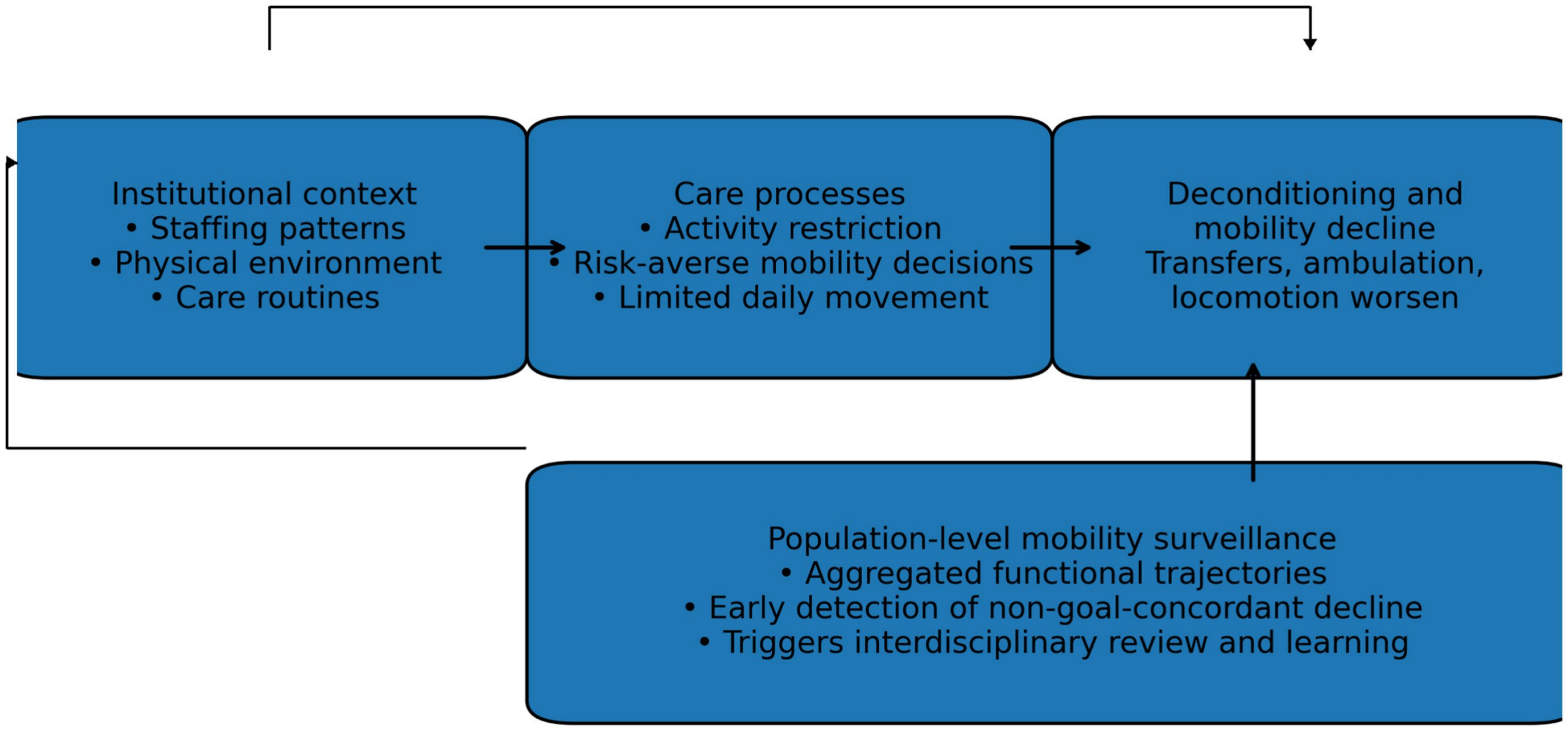
Conceptual framework for mobility surveillance in long-term care, grounded in the Verbrugge and Jette (18) disablement process model and the World Health Organization International Classification of Functioning, Disability and Health (ICF) (19). Both frameworks locate functional decline at the intersection of health conditions and contextual factors. Within institutional long-term care, facility-level context—including staffing, care routines, and environmental constraints—interacts with individual vulnerability to shape mobility trajectories. Population-level surveillance, represented as an intervention point in the framework, applies the logic of epidemiological monitoring to aggregated functional trajectories, enabling early detection of emerging decline patterns and prompting interdisciplinary review before downstream adverse outcomes occur. Bidirectional relationships are represented to reflect how surveillance informs institutional response and how institutional context shapes mobility trajectories.

Institutional factors and care processes contribute to deconditioning and mobility decline. Longitudinal monitoring of functional trajectories provides population-level trend detection that can prompt interdisciplinary review before downstream outcomes occur. This relationship is represented schematically in Figure 1, which illustrates how population-level mobility surveillance functions as an intervention point linking individual trajectories with institutional context.

### Why now: feasibility windows and policy context

A common question is why mobility surveillance has not been implemented already. One explanation is that surveillance systems evolved around events that are straightforward to define and count. Mobility trajectories are inherently longitudinal and require repeated measures, analytic capacity, and careful interpretation. Another explanation is that the primary uses of functional data have historically been documentation, reimbursement, and compliance, rather than population monitoring.

Several changes make feasibility more plausible now. Digital assessment infrastructure has expanded, and standardized instruments such as MDS and CARE are already embedded within workflows (8–10). Value-based payment and quality accountability frameworks increasingly emphasize outcomes that reflect functional status and community living. The COVID-19 era also amplified awareness of deconditioning and the consequences of prolonged inactivity in institutional environments, strengthening the case for monitoring mobility trajectories as an early warning signal.

### Operational implications in long-term care

Implementation barriers must be acknowledged explicitly. Many facilities struggle with assessment accuracy, staffing continuity, and analytic capacity. Therapy teams may be contracted or part-time, and nursing staff operate under substantial workload constraints. Aggregating functional data across assessment periods requires infrastructure that may be uneven across chains and independent facilities.

For this reason, a conservative approach should avoid new reporting mandates. Instead, feasibility-focused functional trend monitoring can be piloted using existing data streams, with centralized analytics performed by health systems, state agencies, or third-party partners where appropriate. The operational goal is not to create another punitive metric. It is to create a disciplined feedback loop for functional trajectories that can support QAPI work and interdisciplinary learning (20).

### International and comparative context

Internationally, functional assessment and trajectory tracking have been operationalized through standardized systems such as interRAI, which has been used across multiple countries and care settings to support person-level assessment and, in some jurisdictions, broader monitoring of functional change (21). While governance structures differ across countries, these examples demonstrate that longitudinal function data can be standardized and used beyond individual documentation. In the United States, the policy question is less whether functional data exist and more whether they are systematically aggregated and interpreted for population-level learning. Additional literature on post-acute care payment evolution, deconditioning, interRAI implementation, frailty, institutional care quality, and recovery trajectories further supports the feasibility and importance of longitudinal mobility monitoring in long-term care settings (26–40).

Analogous initiatives also demonstrate feasibility for monitoring gradual processes. For example, reductions in potentially inappropriate antipsychotic use in nursing homes required longitudinal measurement, attention to risk adjustment, and safeguards against unintended consequences. Over time, the field developed measurement conventions and implementation approaches that balanced accountability with clinical nuance (22, 23). Similar lessons can inform the design of mobility trajectory monitoring programs.

### Equity, ethics, resident goals, and unintended consequences

Equity must be integrated, not appended. Functional trajectories may vary by race, language, socioeconomic status, cognitive impairment, and facility resource availability. Disparities in long-term care quality and access are well documented, and systematic trajectory monitoring has the potential to reveal inequity patterns that are otherwise obscured (24). However, surveillance can also exacerbate inequities if facilities serving higher-acuity populations are penalized without appropriate risk adjustment or if measurement drives selective admissions.

Ethically, surveillance raises questions of privacy, transparency, and dual use. Mobility surveillance should prioritize aggregated reporting, limit identifiability, and be explicit about intended use for improvement rather than punishment, consistent with public health ethics considerations for surveillance systems (11, 12). Just as importantly, mobility preservation is not universally desirable or appropriate. In end-of-life care or advanced progressive disease, reduced mobility may reflect goal-concordant choices prioritizing comfort, pain control, or fatigue management. Surveillance should therefore distinguish unchosen decline from goal-aligned change, and it should encourage documentation of resident goals to support ethical interpretation.

Potential unintended consequences must be named and mitigated. These include gaming through baseline inflation, cherry-picking residents most likely to show gains, risk-averse restriction of activity to avoid documenting decline, and defensive documentation practices. Mitigation strategies include trend-based interpretation rather than punitive thresholds, stratification by hospice and cognitive impairment, and transparent methods for risk adjustment.

### A conservative, feasibility-focused path forward

A conservative implementation pathway is phased. Phase 1 is feasibility. Use existing MDS and CARE data to generate quarterly mobility trajectory summaries at the facility or system level. Phase 2 is interpretation. Embed these summaries in QAPI and interdisciplinary review meetings to identify units or time periods with unexpected decline. Phase 3 is targeted learning. Test practical interventions, such as structured daily mobility prompts, handoffs after therapy discharge, and environment modifications, while monitoring for unintended consequences.

Throughout, risk adjustment should be specified and transparent. At minimum, surveillance should stratify or adjust for baseline mobility, cognitive status, primary diagnosis or impairment category, and hospice or comfort-focused plans. Statistical methods can range from stratified reporting to regression models depending on analytic capacity. The central principle is fairness. Mobility surveillance should not punish complexity. It should create visibility for improvement.

The level at which surveillance should operate depends on infrastructure and accountability goals. Table 1 outlines a tiered model. At the facility level, QAPI teams use internally generated MDS trend reports to compare units, identify time periods of unexpected decline, and guide interdisciplinary review—a function already contemplated in the QAPI program framework (20). At the health system or chain level, central analytics teams aggregate data across facilities to benchmark performance, identify outliers, and target quality improvement resources. At the state or national level, CMS or state agencies incorporate mobility trajectory signals into population monitoring, consistent with the use of other MDS-derived quality measures in public reporting. This tiered structure is not novel: antipsychotic use reduction, pressure injury tracking, and hospitalization monitoring all operate across similar levels. Mobility surveillance can be integrated into existing infrastructure rather than requiring parallel systems (22, 23, 25). Incorporation at the state or national public reporting level would require prior validation work and meaningful stakeholder engagement before formal implementation.

**Table 1:** Tiered model for mobility surveillance implementation in long-term care.

Surveillance level	Primary users	Operational function	Example activities	Primary goal
Facility level	QAPI teams, rehabilitation leadership, nursing leadership	Internal monitoring of mobility trajectories within units or resident populations	Review quarterly MDS trend reports, identify units with unexpected decline patterns, conduct interdisciplinary case reviews, evaluate operational barriers to mobility	Early detection and local quality improvement
Health system or chain level	Corporate clinical leadership, centralized analytics teams	Aggregation and comparison of mobility trends across facilities	Benchmark facilities, identify outlier patterns, allocate quality improvement resources, support targeted operational interventions	System-wide performance learning and support
State or national level	CMS, state agencies, public health entities	Population-level surveillance and policy monitoring	Incorporate mobility trajectory signals into broader quality monitoring frameworks, evaluate regional patterns, inform policy development and research priorities	Public health monitoring and policy guidance

Proposed tiered operational structure for mobility surveillance in long-term care. The framework illustrates how mobility trajectory monitoring could function across facility, health system, and state or national levels using existing functional assessment infrastructure. The model emphasizes surveillance as a population-level monitoring and learning process rather than a punitive performance metric. QAPI, Quality Assurance and Performance Improvement; CMS, Centers for Medicare & Medicaid Services.

### Limitations of this perspective

This perspective has several limitations that should be acknowledged. First, it is conceptual rather than empirical. No new data are presented, and the case for mobility surveillance rests on synthesized evidence and theoretical reasoning rather than original validation studies. Second, the proposed surveillance indicators in Table 2 have not been field-tested for reliability, feasibility, or unintended consequences in real-world long-term care settings. Thresholds for what constitutes actionable decline at the population level would require calibration through prospective implementation research. Third, the framework assumes adequate assessment fidelity in MDS and CARE data; if assessments are conducted inconsistently across facilities or raters, surveillance signals derived from them may reflect documentation variation rather than true functional change. Fourth, risk adjustment specifications proposed here are illustrative; the appropriate covariates and statistical methods for fair facility-level comparisons would require methodological development and stakeholder validation before deployment. These limitations do not undermine the core argument but do underscore that this perspective is a call for structured inquiry rather than a blueprint for immediate implementation.

**Table 2:** Illustrative mobility surveillance indicators.

Indicator	Operational definition	Data source	Timeframe	Interpretation notes
Mobility decline rate	Percentage of long-stay residents with *a* ≥ 2-point decline in MDS locomotion self-performance score between two assessments	MDS 3.0 Section G	Quarterly (approximately 90 days)	Risk-adjust for baseline mobility, cognition, and diagnosis; stratify by long-stay versus post-acute; exclude hospice and comfort-focused care plans; interpret as population-level trend signal
Ambulation loss	Percentage transitioning from walking (with or without assistive device) to wheelchair dependence or bed mobility categories	MDS 3.0 Section G	Quarterly (approximately 90 days)	Exclude hospice and comfort-focused care plans; interpret as population-level trend signal; assess longitudinal trends rather than single-period values
Persistent decline flag	Percentage with decline across two consecutive assessments without recovery to baseline category	MDS 3.0 or CARE	Two consecutive assessments	Use for structured learning review; investigate unit-level patterns, staffing, or environmental constraints; exclude hospice and comfort-focused care plans
Footnotes	Long-stay residents are defined as individuals with length of stay >100 days, consistent with CMS quality measure definitions. MDS 3.0 Section G locomotion items include G0110B (Walk in Room) and G0110C (Walk in Corridor). *a* ≥ 2-point decline represents transition across performance categories and reflects clinically meaningful functional change.			

All indicators are intended for aggregated interpretation and should not be applied to individual-level performance evaluation.

## Conclusion

Long-term care surveillance has improved safety by tracking acute, reportable harms. Yet gradual mobility decline remains a structurally under-monitored trajectory despite its close relationship to outcomes that residents and health systems value. The field already collects functional data through standardized instruments. What is missing is a surveillance lens that aggregates trajectories, supports early detection, and prompts interdisciplinary review.

Integrating mobility trajectories into existing frameworks can be done conservatively. A feasibility-focused, ethically grounded approach can leverage existing assessment data, specify risk adjustment, incorporate resident goals, and guard against punitive use. Mobility surveillance can complement current quality systems by applying similar analytic discipline to functional trajectories as is currently applied to discrete adverse events.

## Data Availability

The original contributions presented in the study are included in the article/supplementary material, further inquiries can be directed to the corresponding author.
